# Tau local structure shields an amyloid-forming motif and controls aggregation propensity

**DOI:** 10.1038/s41467-019-10355-1

**Published:** 2019-06-07

**Authors:** Dailu Chen, Kenneth W. Drombosky, Zhiqiang Hou, Levent Sari, Omar M. Kashmer, Bryan D. Ryder, Valerie A. Perez, DaNae R. Woodard, Milo M. Lin, Marc I. Diamond, Lukasz A. Joachimiak

**Affiliations:** 1 Center for Alzheimer’s and Neurodegenerative Diseases, University of Texas Southwestern Medical Center, Dallas, TX 75390 USA; 2 Molecular Biophysics Graduate Program, University of Texas Southwestern Medical Center, Dallas, TX 75390 USA; 3 Green Center for Molecular, Computational and Systems Biology, University of Texas Southwestern Medical Center, Dallas, TX 75390 USA; 4 Department of Biophysics, University of Texas Southwestern Medical Center, Dallas, TX 75390 USA; 5 Department of Biochemistry, University of Texas Southwestern Medical Center, Dallas, TX 75390 USA

**Keywords:** Prions, Intrinsically disordered proteins

## Abstract

Tauopathies are neurodegenerative diseases characterized by intracellular amyloid deposits of tau protein. Missense mutations in the tau gene (*MAPT*) correlate with aggregation propensity and cause dominantly inherited tauopathies, but their biophysical mechanism driving amyloid formation is poorly understood. Many disease-associated mutations localize within tau’s repeat domain at inter-repeat interfaces proximal to amyloidogenic sequences, such as ^306^VQIVYK^311^. We use cross-linking mass spectrometry, recombinant protein and synthetic peptide systems, in silico modeling, and cell models to conclude that the aggregation-prone ^306^VQIVYK^311^ motif forms metastable compact structures with its upstream sequence that modulates aggregation propensity. We report that disease-associated mutations, isomerization of a critical proline, or alternative splicing are all sufficient to destabilize this local structure and trigger spontaneous aggregation. These findings provide a biophysical framework to explain the basis of early conformational changes that may underlie genetic and sporadic tau pathogenesis.

## Abstract (web summary)

The biophysical mechanisms of how disease-associated tau mutations drive amyloid formation are not well understood. Here the authors use biophysical approaches, cell models and MD simulations and find that the intrinsically disordered repeat domain of tau encodes a metastable local structure and perturbations through mutations and proline isomerization cause an aggregation phenotype in vitro and in cells.

## Introduction

Tauopathies comprise a group of over 20 neurodegenerative diseases in which tau protein aggregates in neurons and glia. Tau aggregation correlates strongly with the degree of dementia and neurodegeneration, especially in Alzheimer’s Disease. The mechanisms by which disease-associated mutations, alternative splicing, or other events promote aggregation and pathology are  not well understood. Understanding the molecular basis of tau aggregation could greatly improve diagnosis and treatment of tauopathies.

**Fig. 1:**
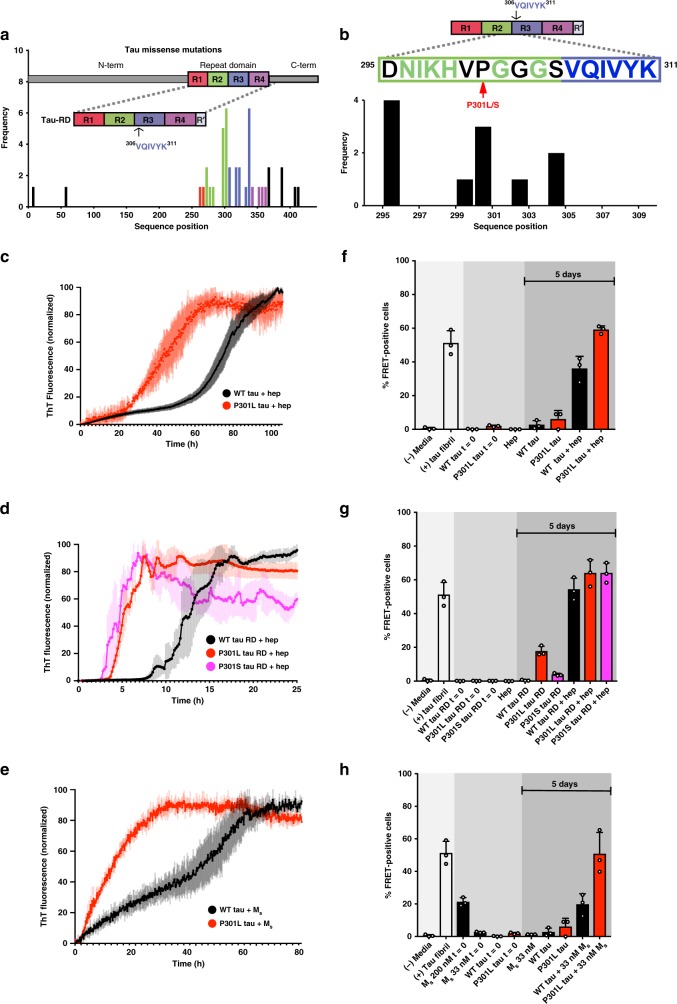
Tauopathy mutations cluster to inter-repeat regions and promote aggregation. **a** Disease-associated mutation frequency found in human tauopathies. Most mutations are found within the repeat domain (tau RD) (repeat 1 = red; repeat 2 = green; repeat 3 = blue; repeat 4 = purple). Amyloidogenic sequence ^306^VQIVYK^311^ is shown in the inset cartoon. **b** Detailed mutation frequencies found near the ^306^VQIVYK^311^ amyloid motif. **c** FL WT tau and mutant P301L tau at a 4.4 µM concentration were mixed with stoichiometric amounts of heparin (4.4 µM), and allowed to aggregate in the presence of ThT at room temperature. Control WT and P301L tau in the absence of heparin yielded no detectible ThT signal change (less than twofold ratio to background signal) over the course of the experiment (see Supplementary Data 1). ThT fluorescence was normalized to the maximum for each condition. **d** WT tau RD and mutant P301L and P301S tau RD at a 4.4 µM concentration were each mixed with equimolar amounts of heparin (4.4 µM), and allowed to aggregate in the presence of ThT at room temperature. Control WT, P301L, and P301S tau RD in the absence of heparin yielded no detectible ThT signal change (less than twofold ratio to background signal) over the course of the experiment (see Supplementary Data 1). **e** WT FL tau and mutant P301L tau at a 4.4 µM concentration were mixed with sub-stoichiometric M_s_ tau seed (33 nM) and allowed to aggregate in the presence of ThT at room temperature. Control WT and P301L tau in the absence of M_s_ yielded no detectible ThT signal change (less than twofold ratio to background signal) over the course of the experiment (see Supplementary Data 1). All ThT experiments were carried out in triplicate. The data are shown as the average with standard deviation and are colored according to mutation. **f**–**h** After 120 h of in vitro incubation, proteins from previous ThT experiments were transduced into tau biosensor cells via lipofectamine (Methods). FRET signal from each condition (tau RD-CFP/tau RD-YFP) was measured by flow cytometry on three biological triplicates of at least 10,000 cells per condition. Error bars represent a 95% CI of each condition

The N-terminal ~ 200 and C-terminal ~ 80 residues of tau are largely disordered, rendering this system refractory to high-resolution studies using structural biology methods^1^. In contrast, the tau repeat domain (tau RD), which spans residues 243–365, is predicted to be more structured^2^, forms the core of amyloid fibrils^3^, and is the minimal region to propagate tau prion strains^4^. Tau RD contains an amyloid motif (^306^VQIVYK^311^) (Fig. 1a) that is central to conversion between the soluble and insoluble states, as it mediates self-assembly, drives amyloid formation in vitro^5^ and promotes pathology in vivo^6^. Nuclear magnetic resonance (NMR) experiments on tau indicate that in solution the ^306^VQIVYK^311^ motif adopts a β-strand conformation^2,7^. Recent cryo-electron microscopy (cryo-EM) studies of tau patient-derived fibrils have shown that ^306^VQIVYK^311^ mediates important contacts in these structures^3,8^. Despite these structural studies, it is not clear how native tau avoids aggregation, nor is it clear how tau transitions from a soluble state to an aggregated assembly.

Polyanions such as heparin, nucleic acids, and arachidonic acid are commonly used to induce tau aggregation in vitro^9–11^. Solution NMR experiments mapped the tau-heparin binding site to repeat 2 just prior to the ^306^VQIVYK^311^ motif, but how this binding event modulates tau aggregation remains unclear^12^. Double electron–electron resonance experiments indicated an expansion of this region upon heparin binding^9^. Cryo-EM structures also suggested an extended conformation of tau when bound to tubulin^13^. Other work mapping the recruitment of molecular chaperones to tau indicated that many chaperones, including Hsp40, Hsp70, and Hsp90, localize around ^306^VQIVYK^311^ ^14^. Furthermore, unfolding of tau RD appeared to promote chaperone binding to the amyloid motif, suggesting that local conformational changes may help recruit factors to limit aggregation^15^. Recent data from our group indicated that soluble monomeric tau exists in at least two conformational ensembles: inert monomer (M_i_), which does not spontaneously self-assemble, and seed-competent monomer (M_s_), which spontaneously self-assembles into amyloid^16^. M_s_ itself adopts multiple stable structures that encode different tau prion strains^17^, which are unique amyloid assemblies that faithfully replicate in living systems. Based on extrapolations, the existence of an aggregation-prone monomer of tau had been previously proposed^18,19^ but our study was the first to biochemically isolate and characterize this species^16^. Different forms of M_s_ have been purified from recombinant protein, and tauopathy brain lysates^16,17^. Using multiple low-resolution structural methods, we have mapped critical structural changes that differentiate M_i_ from M_s_ to near the ^306^VQIVYK^311^ motif and indicated that the repeat two and three region in tau is extended in M_s_, which exposes the ^306^VQIVYK^311^ motif^16^. In contrast, intramolecular disulfide bridge between two native cysteines that flank ^306^VQIVYK^311^ in tau RD is predicted to form a local structure that is incompatible with the formation of amyloid^20^. Thus, conformational changes surrounding the ^306^VQIVYK^311^ amyloid motif appear critical to modulate aggregation propensity. A fragment of tau RD in complex with microtubules hinted that ^306^VQIVYK^311^ forms local contacts with upstream flanking sequence^21^. This was recently supported by predicted models guided by experimental restraints from cross-linking mass spectrometry^16^ and is consistent with independent NMR data^22,23^.

Based on our prior work^16^ we hypothesized that tau adopts a β-hairpin that shields the ^306^VQIVYK^311^ motif and that disease-associated mutations near the motif may contribute to tau’s molecular rearrangement which transforms it from an inert to an early seed-competent form by perturbing this structure. Many of the missense mutations genetically linked to tau pathology in humans occur within tau RD and cluster near ^306^VQIVYK^311^ ^24^ (Fig. 1a, b and Table 1), such as P301L and P301S. These mutations have no definitive biophysical mechanism of action, but are nevertheless widely used in cell and animal models^25,26^. Solution NMR experiments on tau RD encoding a P301L mutation have shown local chemical shift perturbations surrounding the mutation resulting in an increased β-strand propensity^27^. NMR measurements have yielded important insights but require the acquisition of spectra in non-physiological conditions, where aggregation is prohibited. Under these conditions weakly populated states that drive prion aggregation and early seed formation may not be observed^28^.

**Table 1:** List of AlzForum disease-associated mutations

Name	Amino-acid sequence
Tau RD AlzForum Mutations ^a^	R1: 244 QTAPVPMPDLKN-V **K** SK **I** GSTEN **L** KHQP **GG** GK 274 R2: 275 VQII **N** KKLD **LS** N-VQSKCGSKD **N** IKH **VP** G **G** G **S** 305 R3: 306 VQIVYKPVD**LSK**-**V**TSKCGSLGNIHHK**P**GG**GQ** 336 R4: 337 **V** EVKSE **K** LDFKDRVQ **S** KIG **S** LDN **I** TH **VP** G **G** GN 368

^a^Sites of mutation are shown in bold

As with disease-associated mutations, alternative splicing also changes the sequence N-terminal to ^306^VQIVYK^311^. Tau is expressed in the adult brain primarily as two major splice isoforms: three-repeat and four-repeat^29^. The truncated three-repeat isoform lacks the second of four imperfectly repeated segments in tau RD. Expression of the four-repeat isoform correlates with the deposition of aggregated tau tangles in many tauopathies^30^ and non-coding mutations that increase preferential splicing or expression of the four-repeat isoform cause dominantly inherited tauopathies^30–32^. It is not obvious why the incorporation or absence of the second repeat correlates with disease, as the primary sequences, although imperfectly repeated, are relatively conserved.

Previous reports have focused on the structure of a repeat with the assumption that each repeat functions independently within tau RD^33^. These have described a relationship between the length of a repeat fragment, its propensity to spontaneously aggregate, and its seeding capacity in cells^33^. However, inter-repeat interactions may also influence aggregation given that both alternative splicing and many disease-associated mutations cluster around the repeat interfaces (Fig. 1a). Our prior work suggested that wild-type tau aggregates less efficiently because the flanking sequence shields ^306^VQIVYK^311^ ^16^. We hypothesize that the intrinsically disordered tau protein evolved to minimize aggregation by adopting local structure that shields the ^306^VQIVYK^311^ amyloid motif from interactions leading to seed formation and amyloid propagation. We employed an array of in silico, in vitro, and cellular assays to elucidate the molecular interactions and physiological consequences of ^306^VQIVYK^311^ within tau. Our data support a model where disease-associated mutations, alternative splicing, or other factors can destabilize this local structure and expose ^306^VQIVYK^311^ leading to enhanced self-assembly.

## Results

### P301 mutations promote aggregation in vitro and in cells

Missense mutations that change proline 301 to leucine or serine cause dominantly inherited tauopathy^34^ and are associated with neurodegeneration in model systems^26,35^, though the biophysical mechanism is not understood. We studied changes in aggregation propensity driven by mutations at P301 in full-length (FL) tau (2N4R; amino acids 1–441) and tau repeat domain (tau RD; amino acids 244–380) (Supplementary Table 1). First, we monitored aggregation of FL wild-type (WT) tau and mutant (P301L) tau using a Thioflavin T (ThT) fluorescence assay induced with stoichiometric amounts of heparin. We observed that P301L tau (*t*_1/2_ = 41.6 ± 0.5 h) aggregated more rapidly compared with WT tau (*t*_1/2_ = 75 ± 0.3 h) (Fig. 1c and Supplementary Data 1). Next, we compared changes in heparin-induced aggregation of the tau RD, comparing WT, P301L, and P301S mutants. We again observed that the two mutants aggregated faster (P301L tau RD, *t*_1/2_ = 5.2 ± 0.1 h; P301S tau RD, *t*_1/2_ = 3.9 ± 0.1 h) than WT tau RD (WT tau RD, *t*_1/2_ = 12.5 ± 0.2 h) (Fig. 1d and Supplementary Data 1). Consistently, we found that mutations at position 301 (from proline to either leucine or serine) increased aggregation rates by approximately twofold compared with WT in both FL tau and tau RD constructs. Thus, in vitro, tau RD recapitulates key aspects of aggregation observed in FL tau.

The inert conformation of monomeric tau (M_i_) requires co-factors, such as heparin, to spontaneously aggregate in vitro, whereas the seed-competent monomer (M_s_), derived from recombinant protein or Alzheimer’s patient brain material, readily self-assembles to form amyloid^16^. Previously we determined that M_s_ converts FL tau into fibrils at sub-stoichiometric ratios, in contrast to the stoichiometric amounts necessary in heparin-containing reactions^16^. In this study, we evaluated the aggregation propensity of the P301L mutant compared with WT when incubated in the presence of recombinantly produced M_s_. We incubated FL tau with sub-stoichiometric amounts of M_s_ (1:133) and monitored aggregation using ThT. In comparison, we observed that M_s_-seeded P301L tau self-assembled more rapidly (P301L tau, *t*_1/2_ = 8.5 ± 0.6 h) than the WT protein (WT tau, *t*_1/2_ = 40 ± 1.1 h) (Fig. 1e and Supplementary Data 1). P301L tau aggregated faster than WT tau with a fourfold increase in rate after seeding by M_s_. Independent of induction—heparin or M_s_—P301L assembled into ThT-positive aggregates more rapidly. Moreover, tau appeared to be more sensitive to M_s_ seeded aggregation compared with heparin, given the sub-stoichiometric ratios needed for robust aggregation. The effectiveness of M_s_ to seed aggregation of M_i_ may be explained by a direct templating of M_i_ to M_s_ at the amyloid motif region, interface of repeat 2 and 3, which we previously characterized to be more exposed in M_s_^16^. Mutations at the P301 may exacerbate aggregation by unfolding the region surrounding the amyloid motif ^306^VQIVYK^311^, thereby producing a more compatible conformation for the similarly expanded aggregation-prone M_s_ seed.

To test the structural compatibility of aggregates formed by in vitro tau models, we employed tau biosensor HEK293 cells that stably express tau RD (P301S) fused to cyan or yellow fluorescent proteins^25^. These cells sensitively report a fluorescence resonance energy transfer (FRET) signal (tau RD-CFP/tau RD-YFP) only when aggregated in response to tau amyloid seeds, and are unresponsive to aggregates formed by other proteins, such as huntingtin or α-synuclein^36^. Each sample formed amyloid fibril morphologies confirmed by transmission electron microscopy, except for samples not incubated with heparin or M_s_ and the low-concentration M_s_, where no large ordered structures were found (Supplementary Figure 1). The tau biosensor cells responded to FL tau fibrils created by exposure to heparin and showed an increase in seeding activity for the P301L mutant compared with WT fibrils (Fig. 1f and Supplementary Data 2). Next, we compared seeding for the tau RD heparin-induced fibrils and again found that P301L and P301S mutants produced higher seeding activity relative to WT (Fig. 1g and Supplementary Data 2). At last, the seeding activity for the M_s_-induced FL tau fibrils showed a twofold higher activity for P301L compared with WT (Fig. 1h and Supplementary Data 2). WT FL tau and tau RD control samples (no heparin or M_s_) did not produce seeding activity in cells, whereas P301 mutants, both FL and tau RD, showed hints of seeding activity despite not yielding positive ThT signal in vitro (Supplementary Data 1), perhaps owing to the formation of oligomers not captured by ThT. As expected, 33 nM M_s_ control exhibited seeding activity at the onset and did not change after 5 days, but overall signal was low owing to the low concentrations used in the aggregation experiments. Interestingly, WT tau induced with 33 nM M_s_ seeded at similar levels to concentrated control (200 nM) M_s_ samples highlighting efficient conversion of WT tau into seed-competent forms (Fig. 1h and Supplementary Data 2). Thus, P301 mutations promote aggregation in vitro and in cells across different constructs. Importantly, these effects are conserved between FL tau and tau RD.

### Mutations at P301 destabilize native tau structure

**Fig. 2:**
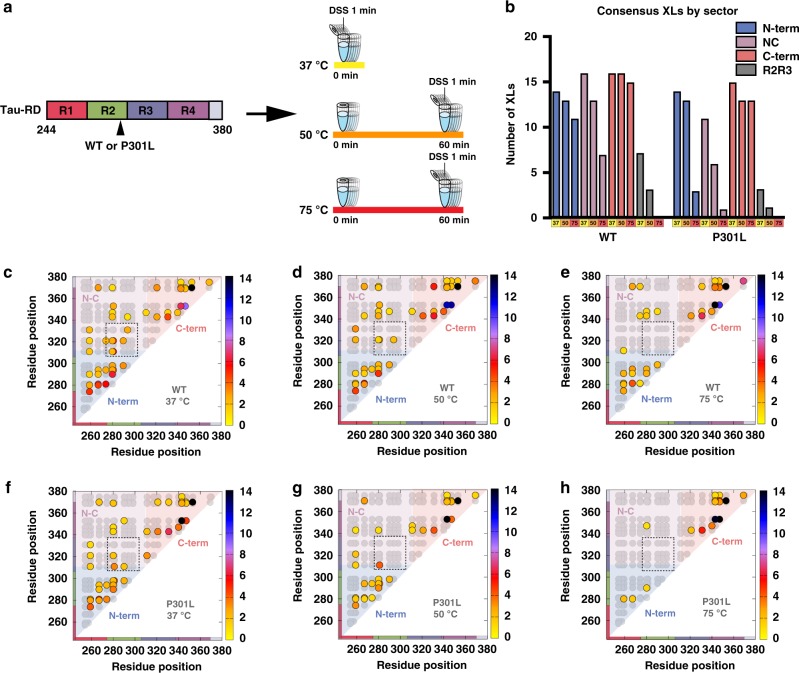
Tau RD encodes global and local structure. **a** Cartoon schematic of tau RD used for XL-MS studies colored according to repeat domain. Recombinant WT and P301L tau RD were heated at 37 °C, 50 °C or 75 °C for 1 hour, then chemically cross-linked using DSS. After cross-linking, trypsin fragmentation, and LC-MS/MS analysis were performed. Each sample was carried out in five technical replicates. **b** Total consensus cross-links parsed by temperature and location in WT and P301L tau RD: within N-terminus (blue; residues 243–310; N-term), within C-terminus (orange; residues 311–380; C-term), span N- and C-terminus (magenta; between residues 243–310 and 311–380; N-C) and between repeat 2 and repeat 3 (R2R3) (gray; between residues 275–305 and 306–336). **c**–**e** Consensus cross-links (circles) are shown in contact maps color coded by average frequency across replicates. The theoretical lysine pairs are shown in the background as gray circles. Cross-link contacts within the N-term (blue), C-term (red), and across N- to C-term (purple) are shown as sectors. The *x* and *y* axis are colored according to repeat number as in Fig. 1. The dashed boxes define inter-repeat cross-links observed between repeat 2 and repeat 3. **f**–**h** Same as **c**–**e** above, except with tau RD that contains a P301L mutation

To determine how the P301L mutation drives conformational changes, we employed cross-linking mass spectrometry (XL-MS) in a heat denaturation experiment. XL-MS defines amino-acid contacts in proteins and can thus guide the determination of structure for large complexes, transient interactions, and dynamics of intrinsically disordered proteins^16,37,38^. To preclude the formation of intermolecular cross-links between monomers, low-concentration samples of WT, P301L, and P301S tau RD (Supplementary Table 1) were incubated at different temperatures, reacted with disuccinimidyl suberate (DSS; a primary amine crosslinker) for 1 min and quenched (Fig. 2a). The cross-linked protein monomers were confirmed by sodium dodecyl sulfate polyacrylamide gel electrophoresis (Supplementary Figure 2a). Cross-linked samples were trypsin digested, analyzed by mass spectrometry and the spectra were searched using Xquest^39^ to identify intramolecular protein contact pairs (Methods and Supplementary Data 3). In each data set, the cross-links reported represent consensus data across five independent samples with a low false discovery rate (FDR) (Methods, Supplementary Figure 3 and Supplementary Data 4). XL-MS of recombinant WT tau RD acquired at 37 °C revealed three clusters of interactions that localize within the N-terminus (residues 243–310; N-term), within the C-terminus (residues 311–380; C-term) and span N- and C-termini (N–C; between residues 243–310 and 311–380) (Fig. 2b, c). Importantly, the experimentally observed cross-links represent only a small subset of all theoretical lysine pairs suggesting that tau RD samples have discrete modes of contacts (Fig. 2c, gray circles). Heating the samples to 50 °C or even 75 °C decreased the number of N-C long-range and N-term short-range contacts identified (Fig. 2b, d, e and Supplementary Figure 2b). The data acquired for WT tau RD in physiological conditions are consistent with a loose metastable structure comprised of weak long-range and short-range contacts that are sensitive to temperature.

In contrast, XL-MS of recombinant tau RD with the P301L mutation revealed an increased susceptibility to heat denaturation. At 37 °C, the cross-links found in P301L tau RD (Fig. 2f) were similar in pattern to WT, except for fewer N–C-terminal long-range contacts (Fig. 2b and Supplementary Figure 2b, c). However, samples incubated at 50 °C and 75 °C revealed a significant reduction in both long-range and short-range contacts in P301L tau RD compared with WT (Fig. 2b). The loss of short-range contacts, both the total number of cross-links and the abundance of each cross-link, were detected particularly within the N-terminal sector, which sits upstream of P301 (Fig. 2b, f–h and Supplementary Figure 2b, c). In contrast, the C-term local contacts sample many theoretical lysine–lysine pairs and remained relatively constant across temperatures for both WT and P301L tau RD, possibly suggesting a higher degree of disorder that is independent of the mutation site. Moreover, a stepwise loss of the inter-repeat cross-links between repeat 2 and 3 was seen in the heat denaturation of WT tau RD and was more pronounced with the P301L mutation, indicating an unfolding of local structure at the interface of repeat 2 and 3, encompassing ^306^VQIVYK^311^ (Fig. 2b, gray bars; Fig. 2c–h, inset box). Thus, although the number of cross-links identified was comparable between WT and P301L tau RD at 37 °C, P301L tau RD retained approximately half as many cross-links as WT when heated. Similar cross-linking profiles were observed for the P301S tau RD sample (Supplementary Figure 2d). Thus, the lack of thermostability in P301 mutated tau RD as compared with WT tau RD suggests that the P301 mutations may lower the threshold for tau to enter into an aggregation-prone conformation.

### Tau RD models show local structure in inter-repeat elements

To gain insight into what types of local structures the inter-repeat elements can form, we used ROSETTA to predict structures of tau RD. We built 5000 models using two parallel strategies in ROSETTA: ab initio that employed fragment libraries derived from experimental structures^40^ and CS-ROSETTA, which leveraged available chemical shifts for tau RD to produce fragment libraries^41^. Both approaches led to a diversity of models consistent with experimentally determined radii of gyration^42^ (Supplementary Figure 4a–d and Supplementary Data 5). Analysis of each structural ensemble showed a propensity to form hairpin-like structures across R1R2, R2R3, R3R4, and R4R’ repeat interfaces centered on the ^271^PGGG^274^, ^301^PGGG^304^, ^333^PGGG^336^, and ^366^PGGG^369^ sequences (Supplementary Figure 4e, Supplementary Figure 5a–d, and 6 and Supplementary Data 5). Previously published solution NMR data have shown that the PGGG sequences in tau can adopt type II β-turns^7^, and the ^301^PGGG^304^ sequence preceding ^306^VQIVYK^311^ is compatible with the formation of a β-hairpin. We illustrated the R2R3 ^306^VQIVYK^311^-containing fragment derived from low energy expanded models produced by each method (Supplementary Figure 4c, d). The ^306^VQIVYK^311^-containing interface has the highest frequency of disease-associated mutations, particularly P301L and P301S (Fig. 1a). Other potential amyloid-forming regions, such as the aggregation-prone ^275^VQIINK^280^ (Supplementary Figure 6), is also preceded by ^271^PGGG^274^ and predicted to form a β-hairpin (Supplementary Figure 4e and Supplementary Figure 5), however, it is absent in recent cryo-EM structures of tau aggregates^3,43^. Mapping known missense mutations onto the ab initio β-hairpin structure at the R2R3 interface (Supplementary Figure 4f), we hypothesized that this cluster of disease-associated mutations could destabilize the β-hairpin secondary structure, thus exposing the amyloid motif ^306^VQIVYK^311^ and enabling aggregation. This model is compatible with recent cryo-EM findings that indicate a disengagement of the ^306^VQIVYK^311^ N-terminal flanking sequence in a fibril structure^3^. Thus, we focused our studies on the R2R3 motif of tau that contains ^306^VQIVYK^311^.

### P301L promotes extended forms of tau

**Fig. 3:**
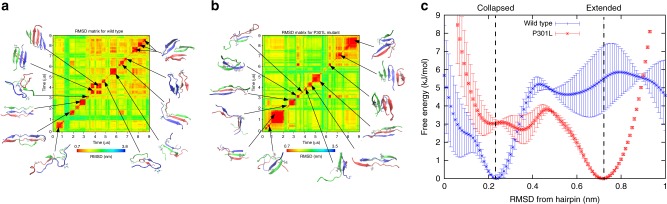
Wild-type and mutant peptides differentially populate collapsed and extended conformations. **a** Trimer conformations obtained from MD simulations of WT peptide fragment (R2R3-WT) with the sequence ^295^DNIKHVPGGGSVQIVYK^311^. Two-dimensional root mean-squared-differences (RMSD’s) are calculated between all pairs of conformations visited during MD simulations. Snapshots of trimeric structures are depicted for selected metastable basins, with each peptide monomer represented by a different color. **b** The same analysis as in **a**, but for the P301L substituted trimer. **c** The free-energy surface as a function of deviation from a canonical hairpin structure. Two distinct basins, corresponding to collapsed and extended sub-ensembles, are found in WT and P301L peptide fragment, respectively. Error bars represent a 95% CI of each RMSD bin

In silico modeling corroborated recent biochemical findings^16^ and suggested a minimal sequence necessary to form a collapsed structure around ^306^VQIVYK^311^. To understand how these structures might self-assemble, we employed molecular dynamics (MD) simulations of two tau peptide fragments comprising the minimally structured fragment centered around the R2R3 interface (^295^DNIKHVPGGGSVQIVYK^311^): R2R3-WT and R2R3-P301L (Supplementary Table 2). To enable sufficient sampling of oligomer structures, we employed an unbiased algorithm based on a recently developed symmetry-constraint approach^44^. The trimer conformations obtained in simulations are depicted on a root mean square deviation (RMSD) matrix for both the R2R3-WT (Fig. 3a) and the R2R3-P301L mutant peptide fragments (Fig. 3b). For the R2R3-WT peptide fragment, we observe a dominant population of trimeric conformations composed of hairpins, whereas the P301L disease-associated mutation stabilizes an extended fibrillar form. The energy basin for the R2R3-WT peptide fragment is predicted to be 5–6 kJ/mol lower in a collapsed state than an extended state, whereas the R2R3-P301L peptide fragment is 3 kJ/mol lower in an extended state than a collapsed state (Fig. 3c and Supplementary Data 6). In addition, the free-energy surface suggests an energy barrier of ~5 kJ/mol to convert the R2R3-WT peptide fragment from collapsed to extended. That same barrier however is <1 kJ/mol for the R2R3-P301L peptide fragment, suggesting a faster rate of kinetic conversion from collapsed hairpin to extended fibrillar form. Thus, MD predicts that the P301L mutation promotes amyloid assembly by destabilizing monomeric hairpin structures.

### Tau amyloid formation is governed by flanking residues

**Fig. 4:**
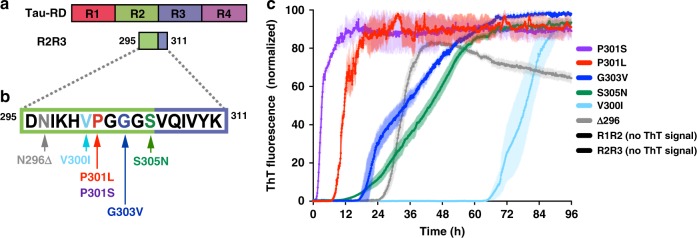
Tauopathy mutations drive aggregation propensity. **a** Schematic of tau RD and the derived peptides representing the minimal structural element around ^306^VQIVYK^311^. **b** WT and mutant peptides were disaggregated, resuspended to 200 µM, and allowed to aggregate in the presence of ThT at room temperature. The WT R2R3 and R1R2 fragment peptides yielded no detectible ThT signal change (less than twofold ratio to background signal) over the course of the experiment (see Supplementary Data 1). ThT signals are shown as average of triplicates with standard deviation, are colored according to mutation and are normalized to the maximum for each condition

**Fig. 5:**
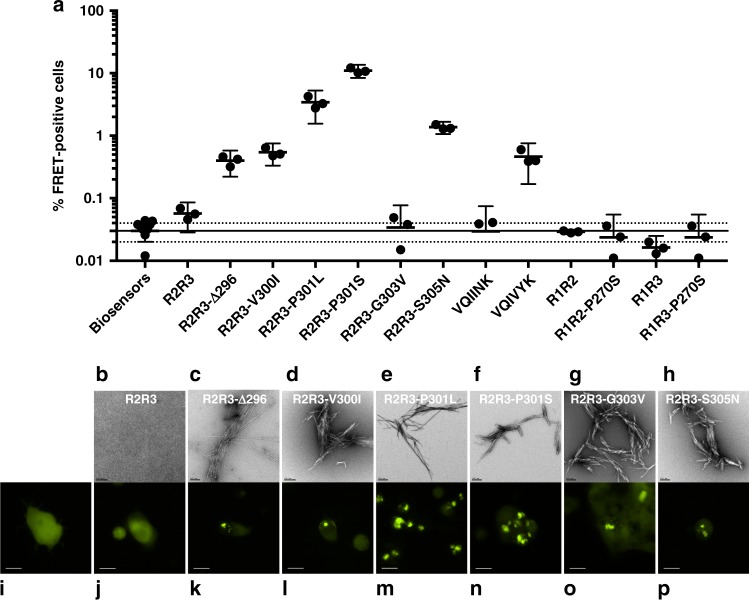
Peptides form amyloid structures and seed in vivo. **a** After 96 h of in vitro incubation, peptides from previous ThT experiments (Fig. 4c) were transduced into tau biosensor cells via lipofectamine (Methods). FRET signal from each condition (tau RD-CFP/tau RD-YFP) was measured by flow cytometry on three biological triplicates of at least 10,000 cells per condition. Error bars represent a 95% CI of each condition. Solid and dashed horizontal lines represent the mean and 95% error from untreated biosensor cells, respectively, for ease of statistical comparison. **b**–**h** Electron microscopy images of each peptide from previous ThT experiments (Fig. 4c). The black bar represents 200 nm distance in each image. **i**–**p** Qualitative fluorescence microscopy images of tau biosensor cells immediately prior to flow cytometry experiments. **i** shows a representative image of untreated biosensor cells**. j**–**p** each shows a representative image of biosensor cells treated with samples from **b**–**h**, respectively. The white bar represents 10 μm distance in each image

In tau RD, ^306^VQIVYK^311^ is necessary for amyloid formation^5,6^. In solution, the ^306^VQIVYK^311^ hexapeptide aggregates spontaneously and rapidly as measured by ThT fluorescence intensity, whereas the upstream N-terminal sequence ^295^DNIKHV^300^ does not aggregate (Supplementary Figure 6). To experimentally test the prediction of a local hairpin structure encompassing ^306^VQIVYK^311^, we employed a mutagenesis study on synthetic peptide systems that recapitulate the minimal hairpin sequence (Supplementary Table 2). Consistent with the prediction from MD simulation, the R2R3-WT peptide fragment did not aggregate readily, with no ThT detected within 96 h (Fig. 4a, c and Supplementary Data 1). By contrast, single disease-associated mutations (Fig. 4b and Supplementary Data 1) substituted into the R2R3 peptide fragment were sufficient to promote spontaneous amyloid formation: R2R3-P301S (*t*_1/2_ = 4.1 ± 1.3 h), R2R3-P301L (*t*_1/2_ = 7.2 ± 0.2 h), R2R3-N296Δ (*t*_1/2_ = 31.9 ± 0.2 h), R2R3-G303V (*t*_1/2_ = 32.1 ± 0.7 h), R2R3-S305N (*t*_1/2_ = 41.2 ± 0.2 h), and R2R3-V300I (*t*_1/2_ = 77.8 ± 1.3 h) (Fig. 4c and Supplementary Data 1). Each of these peptides was confirmed to form amyloid-like fibril morphologies by transmission electron microscopy, except for the WT R2R3 peptide fragment where no large structures were found (Fig. 5b–h).

To test the structural compatibility of peptide aggregates formed by in vitro tau models, we again employed tau biosensor cells^25^. The tau biosensor cells responded to all disease-associated tau peptide fragments that aggregated spontaneously in vitro, but not to the wild-type R2R3 peptide fragment (which did not aggregate in vitro) (Fig. 5a and Supplementary Data 7). Qualitatively, biosensor cells retained their diffused tau localization when untreated or exposed to a wild-type R2R3 peptide fragment but formed fluorescent puncta when cultured with aggregated mutant peptides (Fig. 5i–p). Interestingly, the biosensor cells responded to disease-associated mutant peptides with varying degrees of sensitivity and created distinct aggregate morphologies. This is consistent with amyloid structures that act as distinct templates and form the basis of tau prion strains^4,45^. Thus, the R2R3 peptide fragment model system responds to mutations in vitro and in cells similarly to the FL tau and tau RD system, suggesting that local conformational changes in tau can be recapitulated using shorter fragments.

### Tau splice variants reveal different aggregation propensity

**Fig. 6:**
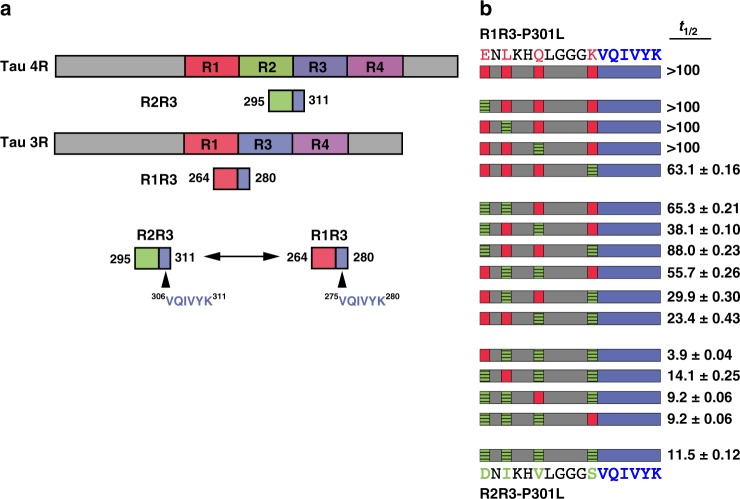
Alternative splicing modulates aggregation propensity. **a** Cartoon schematic for tau 4R and 3R splice isoforms illustrate the difference in primary amino-acid sequence leading into the amyloidogenic ^306^VQIVYK^311^ motif. **b** A full combinatorial panel of R2R3-P301L and R1R3-P301L chimeras were aggregated in vitro. ^306^VQIVYK^311^ is shown in blue, amino acids common between the splice isoforms are shown in gray, amino acids unique to an R3 isoform are colored red, amino acids unique to an R4 isoform are colored green. The aggregation kinetics, represented as *t*_1/2_ in hours with 95% CI, are listed in the right-side column alongside its respective peptide

Tau is expressed in the adult brain as six major splice isoform types that include either three or four repeated segments within RD (Fig. 6a). 3R tau lacks the second of four imperfect repeats. 4R tau correlates strongly with aggregation in most tauopathies^30^ and mutations that increase splicing of the 4R isoform cause dominantly inherited tauopathies^30–32^. We examined whether this splice isoform affects the propensity of ^306^VQIVYK^311^-mediated aggregation owing to the different composition of upstream flanking sequence. We constructed a series of peptide fragments to encompass the R1R3 interface (Fig. 6b). This wild-type peptide fragment R1R3 mimicking a 3R splice isoform did not spontaneously aggregate (Supplementary Figure 7 and Supplementary Data 1). Surprisingly, an R1R3 peptide fragment with a corresponding P301L mutation (R1R3-P270L) also did not aggregate (Fig. 6, Supplementary Figure 7 and Supplementary Data 1). We hypothesized that the R1-leading sequence stabilizes the amyloid motif ^306^VQIVYK^311^, resulting in the aggregation resistance in the presence of disease-associated mutations.

The R1-leading sequence ^264^ENLKHQPGGGK^273^ differs from R2 ^295^DNIKHVPGGGS^304^ at four amino-acid positions. To identify which amino acid(s) governed R1’s stronger inhibitory effects, we constructed 16 peptides with a P301L mutation to represent every combinatorial sequence between the two leading strands and measured their aggregation kinetics (Fig. 6b, Supplementary Figure 7 and Supplementary Data 1). We identified a general trend where the R2R3-P301L peptide fragment aggregates in hours with zero or one R1 substitutions. With two R1 substitutions, the R2R3-P301L peptide aggregation was delayed roughly an order of magnitude to tens of hours. With three R1 substitutions, the R2R3-P301L peptide fragment aggregation was further delayed to hundreds of hours. With all four R1 substitutions in the peptide (R1R3-P301L), no ThT signal was observed within a week (Fig. 6b and Supplementary Figure 7). Thus, all four amino acids contributed to the ability of the R1 leading sequence to delay ^306^VQIVYK^311^-mediated spontaneous aggregation in a 3R splice isoform. This may explain the differential aggregation propensities of tau isoforms in human pathology.

### Stabilizing β-hairpin blocks P301L-mediated aggregation

**Fig. 7:**
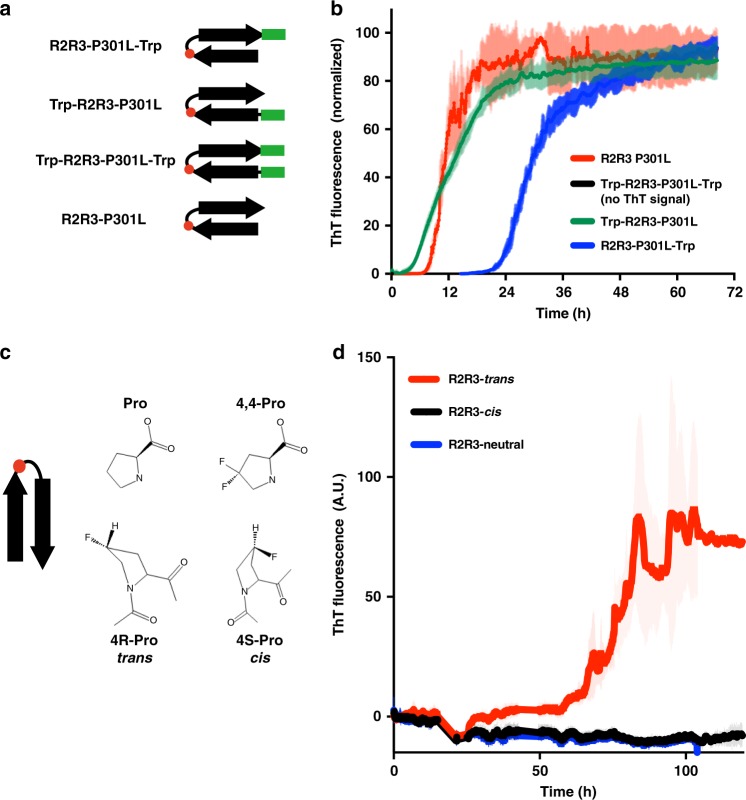
Enhancing β-hairpin structure rescues spontaneous aggregation phenotypes. **a** Cartoon schematic representation of the tryptophan zipper motif (green bar) and controls used to stabilize a β-hairpin structure in an R2R3-P301L peptide fragment (Supplementary Table 2). **b** Aggregation reactions of the tryptophan zipper peptide and controls measured by ThT fluorescence. The Trp-R2R3-P301L-Trp fragment peptide yielded no detectible ThT signal change (less than twofold ratio to background signal) over the course of the experiment (see Supplementary Data 1) ThT signals are shown as average of triplicates with standard deviation and were normalized to the maximum for each condition. **c** Schematic of proline and fluorinated proline analogs used to generate *cis* and *trans* proline conformers at the position corresponding to P301 (red circle) in peptide models. **d** ThT aggregation reactions of the *cis*, *trans*, and neutral proline analogs substituted into the R2R3 peptide fragment. ThT signals are an average of six independent experiments with standard deviation shown

Our model predicted shielding of the ^306^VQIVYK^311^ motif in tau via local β-structure, and thus we hypothesized that artificially stabilizing the termini of this local structure would promote a more inert, closed conformation. To test this, we flanked the R2R3-P301L peptide fragment with a tryptophan zipper (Trp-R2R3-P301L-Trp, Supplementary Table 2 and Fig. 7a), which stabilizes a β-hairpin structure ~ −2.5 to −7 kJ/mol^46^. Consistent with our model, the Trp-R2R3-P301L-Trp peptide fragment does not spontaneously aggregate in vitro (Fig. 7b and Supplementary Data 1).

To ensure that this effect was not a result of adding bulky tryptophan residues, we constructed control peptide fragments that contain only the N-term (Trp-R2R3-P301L) or the C-term (R2R3-P301L-Trp) portion of the tryptophan zipper sequence (Fig. 7a). Both half-sequence controls spontaneously aggregated, implying that a tryptophan at either position is insufficient to block aggregation (Fig. 7b). Only a fully intact tryptophan zipper that stabilizes a β-hairpin conformation ameliorates aggregation propensity. Alternative methods to stabilize a β-hairpin architecture, such as introducing isoelectric interactions, also delayed aggregation: peptides containing two additional aspartic acid on the N-terminus and two lysine on the C-terminus (R2R3-IEZip, Supplementary Table 2) retarded the R2R3-P301L peptide fragment aggregation over an order of magnitude (*t*_1/2_ = 7 h to *t*_1/2_ > 70 h, Supplementary Figure 8 and Supplementary Data 1).

To test this effect in cells, we engineered tau RD (P301S) biosensor cells encoding tryptophan zipper motifs that flank the R2R3 element. These biosensors had a significantly diminished capacity to be seeded; R2R3-P301S peptide fragment aggregates triggered aggregation in 11 ± 1% of tau biosensor cells, but only 0.36 ± 0.12% of the tryptophan zipper stabilized biosensor cells (Supplementary Figure 9 and Supplementary Data 7).

### Proline 301 *cis–trans* isomerization modulates aggregation

Many proteins in the cell utilize proline isomerization as a molecular switch, such as heat shock protein activation^47^ or cell cycle regulation^48^. In some proteins, proline isomerization directly induces or mitigates aggregation into amyloid^49–51^. Proline isomerization events in tau have been proposed to play a role in aggregation and disease^49^, but P301 isomerization has not been linked to tau aggregation and pathology. With the fact that serine or leucine substitutions at P301 proximal to ^306^VQIVYK^311^ drastically alter aggregation propensity, we hypothesized that P301 plays a crucial role inducing a β-turn in a PGGG motif, which mediates a collapsed structure. To test whether isomerization of P301 could influence spontaneous amyloid formation, we constructed a series of R2R3 peptide fragments with proline analogs that preferentially populate either: (1) a *cis* rotamer (2S,4S)-fluoroproline; (2) a *trans* rotamer (2S,4R)-fluoroproline; or (3) an analog that easily interconverts between *cis* and *trans* (4,4)-difluoroproline (Fig. 7c, Supplementary Table 2 and Supplementary Data 1). Only the R2R3-Trans peptide spontaneously aggregated (Fig. 7d and Supplementary Data 1), indicating the potential for proline isomerization events in tau pathogenesis.

## Discussion

Here, we establish the molecular and functional basis for how a series of prominent tau mutations drive aggregation. Integrating experimental and computational approaches, we independently and directly probed the local structural changes within tau. We identified metastable local structures within the inter-repeat junction of tau RD (the repeat 2–3 interface), which encompasses the amyloidogenic ^306^VQIVYK^311^ motif. This R2R3 interface becomes less stable when a disease-associated mutation is present, such as P301L, which is commonly employed in cell and animal models of tauopathy. Thus, P301L and similar mutations decrease the threshold for local structural expansion, especially in the presence of stressors (heat, seeds, heparin, or high concentration). This in turn is predicted to enhance the conversion of tau into a seed-competent form^16^. Thus, the proposed model rationalizes the fundamental molecular mechanisms of aggregation for P301L and at least five other mutations, explains why P301L spontaneously aggregates in animal and cellular models, and defines how splice isoforms of tau and proline isomerization at P301 may contribute to aggregation. Ultimately, these insights may inform the mechanisms of tauopathy in human disease and potential molecular targets for therapeutic development.

In vitro induction of tau aggregation is typically achieved by the addition of polyanionic molecules such as heparin, arachidonic acid, or nucleic acids^10,11,52^. It is thought that heparin binding to tau expands the local conformation of the repeat 2 and repeat 3 regions, thereby exposing amyloidogenic sequences for subsequent aggregation^12,16,52^. This process, however, requires stoichiometric amounts of polyanion and is not a physiological condition, as heparin is not present intracellularly. Our recent work has elucidated a seed-competent form of tau monomer that can promote tau aggregation. This seed-competent monomeric tau is found in AD patient brains and is likely the incipient species contributing to pathology^16^. We find that sub-stoichiometric amounts of M_s_ (1:133) enhance the rate of WT tau aggregation relative to heparin. Parallel experiments with P301L tau show an even more dramatic enhancement. Our data support that the ^306^VQIVYK^311^ motif is preferentially exposed in M_s_ or P301L mutant in contrast to normal tau where it is relatively shielded. Thus, the marked sensitivity of P301L to seeds can be explained by an increased exposure of the aggregation-prone ^306^VQIVYK^311^ sequence. These data suggest that M_s_ functions catalytically to convert normal tau into aggregates. Thus, the proposed seeding mechanism of M_s_ may be generalized to tauopathies that are not caused by mutations.

Ensemble averaging methods, such as NMR, have had limited success in understanding the solution conformations of tau under physiological conditions. They have revealed secondary structure propensities of key regions and proposed the existence of local contacts^2,7,22,23,53^. However, capturing more transient or low population local conformations has been difficult. This is confounded by poor signal to noise, requiring long acquisition times at high concentrations, and non-physiological temperatures to suppress protein aggregation. As such, capturing transient but important local structural signatures have been challenging with classical structural biology methods. Both experiment and simulation have shown that weak local structure may play key roles in limiting aggregation of globular proteins during translation and that these structural elements may play even bigger roles in intrinsically disordered proteins^54,55^. Thus, local structures that bury proximal amyloid sequences may be a general evolutionary design principle that controls aggregation.

Our study has suggested that local structure encompassing the amyloid motif ^306^VQIVYK^311^ regulates aggregation of tau and that the P301L mutation increases susceptibility to conformational changes that expose the ^306^VQIVYK^311^ amyloid motif. Although these differences are subtle, we observe that P301L-mediated structural rearrangements only manifest under moderate stress conditions (i.e., heat, seed). Hence, as compared with NMR, real-time assays, such as XL-MS that kinetically traps conformations are more appropriate to detect metastable sub-populations. These data may explain the elusiveness of a biophysical basis of the cluster of pathogenic mutations near ^306^VQIVYK^311^. Simulations predict that repeat interfaces could encode local structures that are compatible with a β-hairpin and that the P301L mutation, dramatically shifted the equilibrium away from collapsed hairpins to extended fibril-like conformations. Our findings are consistent with published NMR data–PGGG sequences in tau can adopt type II β-turns^7^ and that the P301L mutation increases local β-strand propensity^27^. Thus, our work supports the structural and functional findings that metastable local structures in tau are destabilized by disease-associated mutations.

Guided by our simulations, we predicted that a local fragment spanning the interface between repeat 2 and 3 should encode a minimal structure necessary to replicate this aggregation phenomenon. We examined whether structural perturbations influenced aggregation propensity in a peptide model system that captures this local structural element. The WT tau interface peptide model containing ^306^VQIVYK^311^ did not aggregate spontaneously; however, single point substitutions of six disease-associated mutations immediately N-terminal to ^306^VQIVYK^311^ consistently induced spontaneous aggregation. Given that destabilization of local structure around ^306^VQIVYK^311^ promotes aggregation, stabilizing local structure should rationally mitigate aggregation. By promoting a β-hairpin structure via tryptophan zipper motifs or by using isoelectric forces, a P301L-containing tau peptide had an inhibited propensity to aggregate.

Our data support the hypothesis that local forces are key to preventing aggregation of tau by maintaining specific local structures. Tau is generally considered to be an intrinsically disordered protein, and therefore long-range contacts are unlikely to play a significant role in stability. Published NMR experiments support local structure formation of these regions in tau. Spectra of tau RD (K18; amino acids 244–372) overlaps with a N- and C-terminally expanded tau RD (K32; amino acids 198–394) and even with the splice isoform of tau RD missing repeat 2 (K19; amino acids 244–372 with 275–306 deleted)^7,53^, suggesting that adding residues and even deleting an entire repeat have minimal effects on the local structure. Thus, the conformations of local structures in tau are disproportionally more important to its properties compared with structured proteins. This suggests that peptide fragment models are a valid surrogate and can encapsulate the most relevant endogenous structural elements for investigating aggregation of tau.

Disease-associated mutations found near tau’s amyloid motif, such as P301L or P301S, have no definitive biophysical mechanism but are nevertheless widely used in cell and animal models of disease^25,26^. Using our peptide, tau RD and FL tau model systems, we observe that key mutations dramatically alter aggregation rates on similar time scales in vitro and seed in cell models. We therefore provide an explanation for the toxic-gain-of-function for several mutations.

Previous reports have studied intra-repeat interactions, with the assumption that each repeat functions independently within tau RD^33^. Their peptide models have shown a relationship between the length of a peptide fragment and the seeding capacity of tau to define repeat 3 as the minimal sequence necessary to act as a fully functional seed^33^. Our model defines a minimal regulatory sequence that limits spontaneous aggregation and suggests that inter-repeat structural elements modulate aggregation propensity. The composition of these inter-repeat sequences, governed by missense mutations, directly impacts the stability of local structures and aggregation propensity. It is tempting to speculate that local structure surrounding each of the four inter-repeat regions plays independent roles in the exposure of amyloid sequences. This modular nature of the tau RD region may explain how these independent regions can lead to distinct structures that define tau prion strains^4^. A more comprehensive structure–function analysis of other repeat interfaces may help explain how each inter-repeat element contributes to the formation of different tau structures.

The expression levels of the two major isoform types of tau in the central nervous system—3R and 4R—are similar in the adult brain^29^. However, the 3R:4R ratio of aggregate deposits is disproportionally shifted toward 4R in most tauopathies^30^. Mutations in tau that affect alternative splicing and generate excess 4R isoforms correlate with some genetic tauopathies^31,32^. The N-terminal flanking sequences leading into ^306^VQIVYK^311^ differ by four amino acids between the two isoforms. We find that these two isoforms have drastically different aggregation propensities in the presence of disease-associated mutations (*t*_1/2_ = 7 h vs *t*_1/2_ > 100 h, respectively). Chimeras of R1R3/R2R3 transition from aggregation-resistant to aggregation-prone as they lose R1 N-terminal flanking character. The ability of an R1-leading strand to mitigate ^306^VQIVYK^311^ aggregation may explain why 4R tau correlates more closely with pathology. Thus, inter-repeat contacts may explain aggregation propensities of tau isoforms in disease. Encouraging data for a tau vaccine targeting a ^300^HXPGGG^304^ sequence suggests it is possible to utilize inter-repeat regions to select between pathogenic and non-pathogenic conformations of tau^56^.

Studying the missense mutations in tau has generated valuable disease models^26,35^; however, the majority of human tauopathies have no observed genetic mutation in tau^34^. Critical proline residues N-terminal to the amyloid motif can isomerize into *cis* or *trans* rotamers spontaneously or through unidentified cellular mechanisms. We observe that P301 *cis* and *trans* rotamers have distinct aggregation propensities in vitro. In fact, the aggregation kinetics for a *trans* rotamer of P301 are on par with some well-defined disease mutants (N296Δ, V300I). The concept of proline isomerization triggering aggregation into amyloid is not novel, as this is an accepted mechanism of β2-microglobulin aggregation in kidney dialysis amyloidosis^57^. Other proline residues outside of the tau repeat domain have also been proposed to undergo proline isomerization^49^. Our proposed model suggests a possible mechanism whereby WT tau aggregation could be controlled in vivo: specific prolyl isomerization events—possibly triggered by cellular proline isomerases—could trigger spontaneous aggregation by modulating inter-repeat structural elements.

**Fig. 8:**
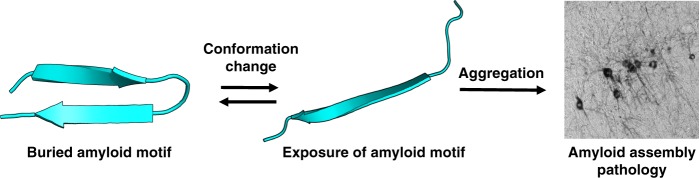
Molecular model of tau amyloid domain structural rearrangement and subsequent aggregation. Naive tau monomer (left) exists with a propensity to form a relatively collapsed structure, which buries the amyloid domain ^306^VQIVYK^311^. In the presence of disease-associated mutations, proline isomerization events, or certain splice isoforms, the equilibrium is shifted to disfavor local compact structure. This exposes the aggregation-prone ^306^VQIVYK^311^ amyloid motif and enhances aggregation propensity, leading to subsequent tau pathology

We propose that sequences N-terminal to tau’s amyloid motif forms local contacts consistent with a β-hairpin-like compact structure. This shields the amyloid motif and mitigates aggregation (Fig. 8). This represents a simple yet comprehensive model of tau aggregation that unifies key observations throughout tau literature. Algorithms that identify potential amyloid-nucleating regions, such as TANGO, have indicated that ~ 75% of aggregation nucleating regions in the human proteome use two or more “gatekeeper” residues, with proline being the most-common single gatekeeping residue^58^. These gatekeeping residues are more likely than average to be the site of disease-associated missense mutations and are consistent with our identification of gatekeeping residues near tau’s amyloid motif. Thus, local flanking sequences and their structural contacts may play an important role in mitigating aggregation propensity in tau and likely other intrinsically disordered proteins. Finally, the identification and characterization of metastable compact structures encompassing ^306^VQIVYK^311^ may itself prove to be a valuable therapeutic target. One might be able to shift the structural rearrangement of tau amyloid motif from exposed (aggregation-prone) to buried (inert) using small molecules, antibodies, or cellular co-factors. Our results indicate that subtle changes in local structure have immense functional ramifications; therefore, small molecules that shift this structural equilibrium modestly may have significant benefits.

## Methods

### Recombinant full-length tau and tau RD production

We utilized several forms of recombinant tau. The pet28b-tau plasmid encoding full-length WT tau was a kind gift from Dr. David Eisenberg (UCLA). The P301L mutation was introduced using QuikChange (Stratagene) with primers shown in Supplementary Table 3. Each plasmid was transformed into BL21-Gold (DE3) cells. Cells were grown in 1 × Terrific Broth media to OD600 1.4 and induced with 1 mM sopropyl β-d-1-thiogalactopyranoside for 3 h at 37 °C. The cells were harvested and lysed in 50 mM Tris, 500 mM NaCl, 1 mM β-mercaptoethanol, 20 mM imidazole, 1 mM phenylmethylsulfonyl fluoride (PMSF), pH 7.5, using an Omni Sonic Ruptor 400 at 4 °C. The lysates were centrifuged, and the supernatant was applied to a Ni-NTA column and eluted with 50 mM Tris, 250 mM NaCl, 1 mM β-mercaptoethanol, 300 mM imidazole. Eluting fractions containing tau were desalted into 50 mM MES, 50 mM NaCl, 1 mM β-mercaptoethanol (pH 6.0) by PD-10 column GE. Exchanged fractions were applied to a HiTrap SP HP (GE) and eluted with a 50 mM–1 M NaCl gradient. Tau containing fractions were concentrated on an Amicon-15 concentrator and applied to a Superdex 200 Increase 10/300 GL (GE) and eluted into 1× PBS (136.5 mM NaCl, 2.7 mM KCl, 10 mM Na_2_HPO_4_, 1.8 mM KH_2_PO_4_, pH 7.4). pNG2-tau RD plasmid encoding tau residues 244–380 was a kind gift from Dr. David Eisenberg (ULCA). The P301L and P301S mutations were introduced using Quikchange (Stratagene) with primers shown in Supplementary Table 3. Tau RD wildtype and mutants were expressed the same way as full-length tau. The cells were harvested and lysed in 1× BRB-80 (80 mM K-PIPES, 1 mM MgSO_4_, 1 mM EGTA, pH 6.8), 0.1% β-ME, 1 mM PMSF, DNAse (5 units/mL from NEB M0303), and RNAse (1 unit/mL from Invitrogen AM2266), using Omni Sonic Ruptor 400 at 4 °C. The lysates were centrifuged, and the supernatant was boiled in a conical tube for 15 min in a water bath. The boiled supernatant was centrifuged at 500 rounds per minute (RPM) for 20 min. The supernatant after centrifugation was filtered using 0.22 µm filter and loaded on HiTrap SP HP (GE) and eluted with a 50 mM–1 M NaCl gradient. Tau RD containing fractions were concentrated on an Amicon-15 concentrator and applied to a Superdex 75 Increase 10/300 GL (GE) and eluted into 1 × PBS (136.5 mM NaCl, 2.7 mM KCl, 10 mM Na_2_HPO_4_, 1.8 mM KH_2_PO_4_, pH 7.4). Aliquots were all stored at − 80 °C in 1 × PBS. Tau seeding monomer (M_s_) was produced as previously described^16^. Specifically, 16 µM WT tau was incubated with heparin (Amsbio) at a 1:1 ratio for 1 h at 37 °C in 1 × PBS. The reaction was resolved on a Superdex 200 Increase 10/300 GL (GE) equilibrated in 1 × PBS. The M_s_ peak eluted at 12 mL, the concentration of the fraction was measured, the sample aliquoted and flash frozen in liquid nitrogen.

### ThT fluorescence aggregation assays

Wild-type or mutant FL tau and tau RD protein was diluted in 1 × PBS with 5 µM β-mercaptoethanol and boiled at 95 °C for 5 min. A final concentration of 4.4 µM heparin (Amsbio) or 33 nM M_s_ seed was added to 4.4 µM tau or tau RD protein in a 60 µL volume mixed with 25 µM ThT and aliquoted into a 96-well clear bottom plate. Peptides were disaggregated as previously described^59^. In brief, peptides were resuspended in a 1:1 mixture (v/v) of TFA (Pierce) incubated at room temperature (RT) for 1 h. In a chemical fume hood, the peptide solution was dried under a stream of nitrogen gas, and then immediately placed under vacuum to remove any residual volatile solvents. The peptide residue was resuspended in 2 × PBS to a 200 µM concentration to adjust the peptide to buffered reaction conditions. In total, 25 µM ThT was added to 200 µL of 200 µM peptide in a 96-well clear bottom plate. All conditions were done in triplicates (except for the R2R3-IEZip experiment) at RT. ThT kinetic scans were run every 5 min on a Tecan M1000 plate reader at 446 nm Ex (5 nm bandwidth), 482 nm Em (5 nm bandwidth). Blank wells containing buffer and ThT were subtracted from experimental values. Samples producing signal to background (ThT only) with ratios only > 2:1 were considered and these values were normalized to the maximum amplitude in each condition. The data were plotted, and the *t*_1/2_ values were derived from a non-linear regression model fitting in GraphPad Prism.

### Transmission electron microscopy

An aliquot of 5 μL of sample was placed onto a glow-discharged Formvar-coated 400-mesh copper grids for 30 s, washed with distilled water, and then negatively stained with 2% uranyl acetate for 1 min. Images were acquired on a Tecnai G^2^ spirit transmission electron microscope (FEI, Hillsboro, OR), serial number: D1067, equipped with a LaB_6_ source at 120 kV using a Gatan ultrascan CCD camera.

### Tau biosensor cells

Biosensor cells were plated into 96-well plates at 20,000 cells per well. For tau and tau RD experiments, after 5 days of incubation with heparin or M_s_, 10 µL of 4.4 µM aggregated protein material was mixed with 1.25 µL lipofectamine and 8.75 µL Opti-MEM, incubated at RT for 30 min, and added to cell media. The “*t* = 0” samples were prepared in the same way but straight from the freezer aliquots. After 2 days, cells were harvested with 0.05% trypsin, then resuspended in Flow buffer (1 × HBSS, 1% FBS, 1 mM ethylenediaminetetraacetic acid (EDTA), 1 × DPBS) and analyzed by flow cytometry. For peptide experiments, 10 µg of aggregated peptide material was added to 0.5 µL lipofectamine and Opti-MEM to a total volume of 10 µL, incubated at RT for 30 min, and added directly to cell media. After 3 days, cells were harvested with 0.05% trypsin, then resuspended in Flow buffer and analyzed by flow cytometry. All conditions were done in triplicates. The Trp Zip biosensor cells expressing the tryptophan zipper motifs flanking the R2R3 element in tau RD were generated as previously described^25^. In brief, the FM5-YFP and FM5-CFP vectors were digested with NdeI (NEB) and ApoI (NEB). The P301L-Trp Zip tau RD fragment was ordered as a geneblock (IDT) (see Supplementary Table 3). Gibson assembly (NEB) was used to insert the fragment into the plasmid. To produce biosenors, HEK293 T cells were plated at a density of 150,000 cells per well in a 24-well dish. The following day, cells were transduced with tau RD P301S Trp Zip CFP or tau RD P301S Trp Zip YFP lentiviral constructs. Cells were grown in virus-containing media for 72 h before expanding. From a 10-cm dish, cells were harvested with 0.05% trypsin, resuspended in flow cytometry buffer (HBSS plus 1% FBS and 1 mM EDTA), and subjected to FACS (Sony Biotechnology). Populations of CFP and YFP dual-positive cells with a CFP:YFP median fluorescent intensity (MFI) ratio of 1:3.7 (standardized to their relative brightness) were selected to yield a FRET donor:acceptor molar ratio of 1:1. CFP or YFP single-positive cells with an equivalent MFI to dual-positive cells were selected. Following FACS and expansion, single-positive cells were maintained and used as a polyclonal line. Dual-positive cells were used to generate monoclonal lines. Here, cells were plated sparsely in a 10-cm dish and allowed to expand for 10 d, at which time cloning cylinders (Bel-Art Products) were used to isolate single clones. All stable cell lines were amplified, frozen down, and stored in liquid nitrogen until use. The derived monoclonal biosensor cell lines were empirically tested for best FRET signal to noise, and the same monoclonal cell line was used for all experiments.

### Flow cytometry

A BD LSRFortessa was used to perform FRET flow cytometry. To measure CFP and FRET, cells were excited with the 405 nm laser, and fluorescence was captured with a 405/50 nm and 525/50 nm filter, respectively. To measure YFP, cells were excited with a 488 laser and fluorescence was captured with a 525/50 nm filter. To quantify FRET, we used a gating strategy where CFP bleed-through into the YFP and FRET channels was compensated using FlowJo analysis software. The MACSQuant VYB (Miltenyi) was used to perform FRET flow cytometry. To measure CFP and FRET, cells were excited with the 405 nm laser, and fluorescence was captured with a 405/50 nm and 525/50 nm filter, respectively. To measure YFP, cells were excited with a 488 laser and fluorescence was captured with a 525/50 nm filter. To quantify FRET, we used a gating strategy similar to that previously described. In brief, CFP bleed-through into the YFP and FRET channels was compensated using MACSQuantify Software from Miltenyi Biotec. Because some YFP-only cells exhibit emission in the FRET channel, we introduced and additional gate to exclude from analysis cells that exert a false-positive signal in the FRET channel (i.e., false FRET gate). Subsequently, we created a final bivariate plot of FRET vs. CFP and introduced a triangular gate to assess the number of FRET-positive cells. This FRET gate was adjusted to biosensor cells that received lipofectamine alone and are thus FRET-negative. This allows for direct visualization of sensitized acceptor emission arising from excitation of the CFP donor at 405 nm. The integrated FRET density, defined as the percentage of FRET-positive cells multiplied by the median fluorescence intensity of FRET-positive cells, was used for all analyses. For each experiment, 20,000 cells per replicate were analyzed and each condition was analyzed in quadruplicate. Data analysis was performed using FlowJo v10 software (Treestar).

### XL-MS sample preparation and mass spectrometry

Preparation of tau RD was cross-linked at a total protein concentration of 1.0 mg/mL using 100 µg of starting material. The cross-linking buffer was 1 × PBS with 3 mM DTT. Five replicates for each condition (37 °C, 50 °C, and 75 °C) were prepared. Samples for 50 °C and 75 °C conditions were equilibrated at the appropriate temperature for 1 h before cross-linking. The cross-linking reaction was initiated by adding DSS stock solution (25 mM DSS-d_0_ and -d_12_, Creative Molecules) in DMF to a final concentration of 1 mM. Samples were further incubated at 37 °C, 50 °C, or 75 °C for 1 min with 350 RPM shaking. Excess reagent was quenched by addition of Tris (pH 7.5) to 100 mM and incubation at 37 °C for 30 min, and subsequently flash frozen by liquid nitrogen and evaporated to dryness by lyophilization. Proteins were resuspended in 8 M urea, reduced with 2.5 mM TCEP (37 °C, 30 min) and alkylated with 5 mM iodoacetamide (30 min, RT, protected from light). The sample solutions were diluted to 1 M urea with 50 mM ammonium hydrogen carbonate and trypsin (Promega) was added at an enzyme-to-substrate ratio of 1:50. Proteolysis was carried out at 37 °C overnight followed by acidification with formic acid to 2% (v/v). Samples were then purified by solid-phase extraction using Sep-Pak tC18 cartridges (Waters) according to standard protocols. Samples were evaporated to dryness and reconstituted in water/acetonitrile/formic acid (95:5:0.1, v/v/v) to a final concentration of ~ 0.5 µg/µL. In total, 2 µL each were injected for duplicate LC-MS/MS analyses on an Eksigent 1D-NanoLC-Ultra HPLC system coupled to a Thermo Orbitrap Fusion Tribrid system. Peptides were separated on self-packed New Objective PicoFrit columns (11 cm × 0.075 mm I.D.) containing Magic C_18_ material (Michrom, 3 µm particle size, 200 Å pore size) at a flow rate of 300 nL/min using the following gradient. 0–5 min = 5% B, 5–95 min = 5–35% B, 95–97 min = 35–95% B and 97–107 min = 95% B, where A = (water/acetonitrile/formic acid, 97:3:0.1) and B = (acetonitrile/water/formic acid, 97:3:0.1). The mass spectrometer was operated in data-dependent mode by selecting the five most abundant precursor ions (*m/z* 350–1600, charge state 3+ and above) from a preview scan and subjecting them to collision-induced dissociation (normalized collision energy = 35%, 30 ms activation). Fragment ions were detected at low resolution in the linear ion trap. Dynamic exclusion was enabled (repeat count 1, exclusion duration 30 s).

### Analysis of mass spectrometry data

Thermo.raw files were converted to the open.mzXML format using msconvert (proteowizard.sourceforge.net) and analyzed using an in-house version of xQuest^50^. Spectral pairs with a precursor mass difference of 12.075321 Da were extracted and searched against the respective FASTA databases containing tau (TAU_HUMAN P10636-8) or with a P301L/S substitution. xQuest settings were as follows: Maximum number of missed cleavages (excluding the cross-linking site) = 2, peptide length = 5–50 aa, fixed modifications = carbamidomethyl-Cys (mass shift = 57.021460 Da), mass shift of the light crosslinker = 138.068080 Da, mass shift of mono-links = 156.078644 and 155.096428 Da, MS^1^ tolerance = 10 ppm, MS^2^ tolerance = 0.2 Da for common ions and 0.3 Da for cross-link ions, search in ion-tag mode. Post-search manual validation and filtering was performed using the following criteria: xQuest score > 25, mass error between − 2.2 and + 3.8 ppm, %TIC > 10, and a minimum peptide length of six aa. In addition, at least four assigned fragment ions (or at least three contiguous fragments) were required on each of the two peptides in a cross-link. FDRs for the identified cross-links were estimated using xprophet^60^ and estimated to be 1.3–10% (Supplementary Figure 3, Supplementary Data 2). At each temperature, the five replicate data sets were compared and only cross-links present in five of the five data sets were used to generate a consensus data set (Supplementary Data 3). Cross-link data with information of cross-linked residue positions and nseen (frequency) were visualized using customized gnuplot scripts.

### Model generation of tau RD using ROSETTA

The backbone NH, N, CA, CB, and C=O chemical shift assignments for the tau fragment from 243–368 (bmrbId = 19253) were used in CS-Rosetta to generate fragment libraries for subsequent model refinement^61^. First, chemical shift parameters were used to predict backbone torsional angles using TALOS to generate a CS-guided fragment library representing the conformations of the protein^61^. For the ab initio ROSETTA calculations, the tau RD sequence was used to generate 3-mer and 9-mer fragments derived from the protein data bank using the fragment picker tool^40^. The Rosetta energy function was used to assemble and iteratively refine 5000 structural models using each set of fragments^40,41,62^ (Supplementary Data 5). Cα-based RMSDs were computed in Rosetta for tau RD and hairpin fragments (Supplementary Data 5). Clustering analysis of the tau RD ensemble showed similar results yielding median RMSDs of 19.5 Ang^2^ and 19.75 Ang^2^ for ab initio and CS-ROSETTA simulations, respectively (Supplementary Data 5). Ensemble wide calculation of cα–cα end to end distances between residues 264–280, 295–311, 327–343 and 359–375 were carried out using a python script (Supplementary Data 5). All simulations were performed on UTSW’s biohpc computing cluster. All plots were generated with gnuplot. Images were created using Pymol.

### Peptide synthesis

All peptides were synthesized as ordered by Genscript with N-terminal acetylation and C-terminal amidation modifications. Peptides were purified to > 95% purity by FPLC via an Agilent ZORBAX StableBond 250 mm C8 column.

### Molecular dynamics simulations

Well-Tempered Metadynamics^63^ was employed to enable accelerated conformational sampling and to construct the associated free energy surface. Metadynamics was performed on a two-dimensional space of parallel-β sheet content and anti-parallel sheet content. To increase search efficiency in oligomeric space, we have incorporated conformational symmetry constraints, which have been shown to enable sampling of multi-polymer landscapes^44^. The initial dodecahedron simulation box was constructed from a trimer of a randomly unfolded structure of 295–311 by adding 7587 SPCE explicit waters and three neutralizing Cl ions (one for each monomer). The AMBER99sb-ildn force-field^64^ was used for all simulations. After an initial 1009 steepest descent steps of converged energy minimization, 10 ns of NVT and 20 ns of NPT (first 10 with Berendsen^65^ and the last 10 with Parrinello-Rahman^66^ barostats) equilibrations were performed. The subsequent production level trajectories are based on 5 fs time steps using hydrogen-only virtual sites^67^. Production level trajectories were obtained for an NPT ensemble with Parrinello-Rahman barostat, and periodic boundary conditions with Particle Mesh Ewald (PME)^68^ summation for long-range electrostatics. The tuned well-tempered metadynamics parameters are 10, 1.4 kJ/mol, and 0.3 for bias factor, Gaussian height, collective variable space Gaussian widths, respectively. The Gaussian perturbations were included into MD every 2.5 ps using the PLUMED package^69^ as an external patch to Gromacs-5.0.4^70^. A total of 18 μs trajectories were generated, 9 μs for wildtype and 9 μs for the P301L mutant, over a total of six independent runs. All simulations were done on UTSW’s bioHPC computing cluster.

### Statistics

All statistics were calculated using GraphPad Prism 8.0. Three independent ThT experiments were run for each condition. The data were normalized to the highest amplitude and averages and standard deviations were plotted. Plots were fitted to a non-linear regression model, from which *t*_1/2_ values were derived. *t*_1/2_ error represents a 95% CI. Flow cytometry cell aggregation was conducted in three independent experiments, whose values are plotted. Error bars represent a 95% CI.

### Reporting summary

Further information on research design is available in the Nature Research Reporting Summary linked to this article.

## Supplementary information

Supplementary Information

Description of Additional Supplementary Files

Supplementary Data 1

Supplementary Data 2

Supplementary Data 3

Supplementary Data 4

Supplementary Data 5

Supplementary Data 6

Supplementary Data 7

Reporting Summary

## Data Availability

ThT data for tau, tau RD, and peptide experiments is available in Supplementary Data 1. Raw biosensor FRET analysis for tau and tau RD seeding experiments is available in Supplementary Data 2. Raw cross-linking mass spectrometry data are available in Supplementary Data 3 and 4. Summary of ab initio and CS-ROSETTA ensemble and hairpin analysis available in Supplementary Data 5. Summary of MD trajectory energetics are available in Supplementary Data 6. Raw biosensor FRET analysis for synthetic peptide-seeding experiments is available in Supplementary Data 7. Other supporting data available upon reasonable request from the authors.

## Code availability

All tau RD simulations were carried out with CS-ROSETTA and ROSETTA (available at https://www.rosettacommons.org/). All peptide MD simulations were performed using Gromacs-5.0.4 (available at http://www.gromacs.org). The metadynamics runs are carried out with the PLUMED-2.1.2 interface to Gromacs (available at http://www.plumed.org)
