# Supplementary material for: Tau local structure shields an amyloid-forming motif and controls aggregation propensity
Source: Nat Commun. 2019 Jun 7;10:2493. doi: 10.1038/s41467-019-10355-1 (PMC6555816; doi:10.1038/s41467-019-10355-1)
Supplement: Supplementary file 2 — Description of Additional Supplementary Files [file 41467_2019_10355_MOESM2_ESM.pdf]

Description of Additional Supplementary Files

File name: Supplementary Data 1

Description: Summary of ThT experimental values for tau, tau RD and peptide aggregation experiments.

File name: Supplementary Data 2

Description: Summary of P301S FRET biosensor seeding values for tau and tau-RD aggregates. Data is shown as % FRET positive cells.

File name: Supplementary Data 3

Description: Summary of technical replicate XL-MS data for WT, P301L and P301S tau RD at 37ºC, 50ºC and 75ºC.

File name: Supplementary Data 4

Description: Summary of consensus crosslink pairs for WT, P301L and P301S tau RD at 37ºC, 50ºC and 75ºC.

File name: Supplementary Data 5

Description: Summary of ab initio and CS ROSETTA ensemble and hairpin analysis.

File name: Supplementary Data 6

Description: Summary and RMSD and energetics of WT and P301L peptide MD trajectories.

File name: Supplementary Data 7

Description: Summary of P301S FRET biosensor seeding values for peptide aggregates. Summary of P301S and P301L TrpZip biosensor seeding values for R2R3 P301S peptide aggregates. Data is shown as %FRET positive cells.
