# Supplementary material for: Tau local structure shields an amyloid-forming motif and controls aggregation propensity
Source: Nat Commun. 2019 Jun 7;10:2493. doi: 10.1038/s41467-019-10355-1 (PMC6555816; doi:10.1038/s41467-019-10355-1)
Supplement: Supplementary file 10 — Reporting Summary [file 41467_2019_10355_MOESM10_ESM.pdf]

nature research

Corresponding author(s): Lukasz A. Joachimiak

Last updated by author(s): Apr 26, 2019

Reporting Summary

Nature Research wishes to improve the reproducibility of the work that we publish. This form provides structure for consistency and transparency in reporting. For further information on Nature Research policies, see Authors & Referees and the Editorial Policy Checklist.

Statistics

For all statistical analyses, confirm that the following items are present in the figure legend, table legend, main text, or Methods section.

n/a	Confirmed	
☐	☒	The exact sample size (*n*) for each experimental group/condition, given as a discrete number and unit of measurement
☐	☒	A statement on whether measurements were taken from distinct samples or whether the same sample was measured repeatedly
☒	☐	The statistical test(s) used AND whether they are one- or two-sided<br>*Only common tests should be described solely by name; describe more complex techniques in the Methods section.*
☒	☐	A description of all covariates tested
☒	☐	A description of any assumptions or corrections, such as tests of normality and adjustment for multiple comparisons
☒	☐	A full description of the statistical parameters including central tendency (e.g. means) or other basic estimates (e.g. regression coefficient) AND variation (e.g. standard deviation) or associated estimates of uncertainty (e.g. confidence intervals)
☒	☐	For null hypothesis testing, the test statistic (e.g. *F*, *t*, *r*) with confidence intervals, effect sizes, degrees of freedom and *P* value noted<br>*Give P values as exact values whenever suitable.*
☒	☐	For Bayesian analysis, information on the choice of priors and Markov chain Monte Carlo settings
☒	☐	For hierarchical and complex designs, identification of the appropriate level for tests and full reporting of outcomes
☒	☐	Estimates of effect sizes (e.g. Cohen's *d*, Pearson's *r*), indicating how they were calculated

Our web collection on statistics for biologists contains articles on many of the points above.

Software and code

Policy information about availability of computer code

Data collection	All tau-RD simulations were carried out with CS-ROSETTA and ROSETTA (available at https://www.rosettacommons.org/). All peptide molecular dynamics simulations were performed using Gromacs-5.0.4 (available at http://www.gromacs.org). The metadynamics runs are carried out with the PLUMED-2.1.2 interface to Gromacs (available at http://www.plumed.org)
Data analysis	All images of structures were produced in pymol 1.8.4.2. All ThT curves were generated with GraphPad Prism 7. Data analysis of FACS data was carried out in Flojo 9.0.5. XL-MS data analysis was carried out using Xquest V2.1.3 (available at http://proteomics.ethz.ch/cgi-bin/xquest2_cgi/index.cgi). All XL-MS plots were generated with gnuplot 5.2. CD data were fit using the bestsel software (http://bestsel.elte.hu/). Analysis of the data produced by molecular dynamics simulations were done using standard internal analysis tools of Gromacs-5.0.4 and PLUMED-2.1.2. Gromacs is available at http://www.gromacs.org while PLUMED is available at http://www.plumed.org. Chemical structures of prolines were built using Chemdraw 17.1.

For manuscripts utilizing custom algorithms or software that are central to the research but not yet described in published literature, software must be made available to editors/reviewers. We strongly encourage code deposition in a community repository (e.g. GitHub). See the Nature Research guidelines for submitting code & software for further information.

Data

Policy information about availability of data

All manuscripts must include a data availability statement. This statement should provide the following information, where applicable:

- Accession codes, unique identifiers, or web links for publicly available datasets
- A list of figures that have associated raw data
- A description of any restrictions on data availability

Raw Thioflavin T aggregation data is available in Supplementary Data 1. FRET biosensor analysis of tau and tauRD is available in Supplementary Data 2. Raw crosslinking mass spectrometry data is available in Supplementary Data Table 3 and 4. Rosetta hairpin analysis is available in Supplementary Data Table 5. MD simulation analyses are available in Supplementary Data Table 6. FRET biosensor analysis of synthetic peptides is available in Supplementary Data 7. Other

supporting data available upon reasonable request from the authors.

Field-specific reporting

Please select the one below that is the best fit for your research. If you are not sure, read the appropriate sections before making your selection.

☒ Life sciences ☐ Behavioural & social sciences ☐ Ecological, evolutionary & environmental sciences

For a reference copy of the document with all sections, see nature.com/documents/nr-reporting-summary-flat.pdf

Life sciences study design

All studies must disclose on these points even when the disclosure is negative.

Sample size

We did not study populations either of animals or humans, thus sample size is not applicable. In the case of Rosetta simulations, we arbitrarily chose 5000 as the number of simulations to test. This is relatively standard in the field and size of the protein. Using our computer cluster this represented 2 weeks of computer time.

Data exclusions

No data were excluded in the analyses

Replication

Figure 1. ThT experiments were performed as three biological replicates, and reported as averages with standard deviations and available in Supplementary Data 1. Biosensor FRET assay was performed as three biological replicates and reported as averages with standard deviations and available in Supplementary Data 2.

Figure 2. XL-MS experiments were carried out as five technical replicates to derive accurate consensus pairs for WT and P301L tau-RD at 37oC 50oC and 75oC. Raw XL-MS data is reported in Supplementary Data 3 and 4.

Figure 3. Molecular dynamics simulations for WT and P301L mutant peptides were carried out as three independent trajectories to confirm convergence. The energetics and conformational rmsd was performed as three replicates and reported as averages with standard deviations. Energetic analysis of MD trajectories is reported in Supplementary Data 5.

Figure 4. ThT experiments were performed as three biological replicates with three technical triplicates in each replicate, and reported as averages and raw data is available in Supplementary Data 1.

Figure 5. FRET biosensor experiments were carried out as three biological replicates for each condition. For each replicate, 10,000 single cells were measured and counted towards data analysis. TEM images were collected on two separate samples and a representative image is shown. Raw FRET biosensor data is in Supplementary Data 7.

Figure 6. ThT experiments on splicing variant peptides were performed as three biological replicates and as T1/2max with standard deviation. Raw data is available in Supplementary Data 1.

Figure 7. ThT experiments on Trp-Zip peptides were performed as three biological replicates and reported as average curves with standard deviations. ThT experiments on peptides with proline analogs were carried out as six technical replicates and reported as average curves with standard deviations and available in Supplementary Data 1.

Figure 8. N/A

Supplementary Figure 1. TEM images were collected on two separate samples and a representative image is shown.

Supplementary Figure 2. Representative gels for the crosslinked WT, P301L and P301S tau-RD are shown. Histogram for sum of consensus pairs identified for WT, P301L and P301S are shown, raw data available in Supplemental Data 2 and 3. XL-MS experiments were carried out as five technical replicates to derive accurate consensus pairs for P301S tau-RD at 37oC 50oC and 75oC. Raw XL-MS data is reported in Supplementary Data 3 and 4.

Supplementary Figure 3. Representative true positive and false positive distribution plots for each of the datasets to illustrate how false discovery values for each dataset are derived. Raw data is available in Supplementary Data 3.

Supplementary Figure 4. 5000 models were built for Rosetta ab initio and CS-ROSETTA simulations of tau RD which is typical for this length of protein. Ensemble-wide alignment of models shows conformational heterogeneity. Alignment of idealized hairpins to ensembles reveals a proportion of structures contain hairpins. Raw data is available in Supplementary Data 5.

Supplementary Figure 5. Distance-based analysis of models reveals a significant fraction of models contain collapsed structures at inter-repeat regions. Raw data is available in Supplementary Data 5.

Supplementary Figure 6. ThT experiments on hairpin fragments were performed as three biological replicates and reported as average curves with standard deviation. The raw ThT data is available in Supplementary Data 1.

Supplementary Figure 7. ThT experiments on splicing variant peptides were performed as three biological replicates and reported as averages with standard deviations. Raw data is available in Supplementary Data 1.

Supplementary Figure 8. ThT experiments on Alt-Zip peptides were performed as a single experiment. Raw data is available in Supplementary

Data 1.

Supplementary Figure 9. FRET biosensor measurements comparing tau-RD P301S and tau-RD P301L Trpzip biosensor cells lines were carried out as biological triplicates. Data is available in Supplementary Data 7.

Randomization	N/A
Blinding	N/A

Reporting for specific materials, systems and methods

We require information from authors about some types of materials, experimental systems and methods used in many studies. Here, indicate whether each material, system or method listed is relevant to your study. If you are not sure if a list item applies to your research, read the appropriate section before selecting a response.

Materials & experimental systems

n/a	Involved in the study	
☒	☐	Antibodies
☐	☒	Eukaryotic cell lines
☒	☐	Palaeontology
☒	☐	Animals and other organisms
☒	☐	Human research participants
☒	☐	Clinical data

Methods

n/a	Involved in the study	
☒	☐	ChIP-seq
☐	☒	Flow cytometry
☒	☐	MRI-based neuroimaging

Eukaryotic cell lines

Policy information about cell lines

Cell line source(s)	Cell line (HEK293) Tau RD P301S FRET Biosensor, ATCC CRL-3275
Authentication	Cell lines were not authenticated
Mycoplasma contamination	Cells were confirmed to be free of mycoplasma contamination
Commonly misidentified lines (See ICLAC register)	*Name any commonly misidentified cell lines used in the study and provide a rationale for their use.*

Flow Cytometry

Plots

Confirm that:

☐ The axis labels state the marker and fluorochrome used (e.g. CD4-FITC).

☐ The axis scales are clearly visible. Include numbers along axes only for bottom left plot of group (a 'group' is an analysis of identical markers).

☐ All plots are contour plots with outliers or pseudocolor plots.

☒ A numerical value for number of cells or percentage (with statistics) is provided.

Methodology

Sample preparation	HEK293 tau biosensor cells expressing tau-CFP and tau-YFP, as previous reported.
Instrument	BD LSRFortessa
Software	Flojo 9.0.5
Cell population abundance	All single cells, which represented >80% of all particles sorted, were counted towards the final analysis.
Gating strategy	Gating strategies have been previous described and cited in the article (Holmes 2014).

☐ Tick this box to confirm that a figure exemplifying the gating strategy is provided in the Supplementary Information.
